# Immune checkpoint inhibitors-related cytokine storm syndrome: a systematic review of characteristics, treatment, and outcomes

**DOI:** 10.3389/fimmu.2026.1860219

**Published:** 2026-07-30

**Authors:** Qiong Jin, Qinglei Fu, Bifeng Fei

**Affiliations:** Huzhou Wuxing District People’s Hospital, Huzhou Wuxing District Maternity and Child Health Hospital, Huzhou, China

**Keywords:** immune checkpoints, immune-related adverse events, literature analysis, case reports, cytokine release syndrome

## Abstract

**Objective:**

To analyze and summarize the general patterns and key points of treatment for cytokine release syndrome (CRS) induced by immune checkpoint inhibitors (ICIs), and provide references for the differential diagnosis and treatment of this immune-related adverse event (irAE) and the safe application of ICIs.

**Methods:**

Case reports published in domestic and international databases were collected. Basic information of patients and CRS were extracted from the included cases to clarify the characteristics and intervention measures of this irAE.

**Results:**

A total of 58 articles involving 59 patients were finally included, comprising 36 males and 23 females. The average age of the included patients was (58.54 ± 15.29) years, and lung cancer was the most common primary disease. Ten types of ICIs were used during treatment, and CRS occurred within the first four cycles of ICI administration in the vast majority of patients. Abnormal biochemical and inflammatory indicators could contribute to the diagnosis and confirmation of CRS, while the initial clinical manifestations of included patients were mostly non-specific, with about half of them experiencing at least one other concurrent irAE. With active treatment, 50 patients had a good outcome, but 9 patients died.

**Conclusion:**

CRS induced by ICIs is a rare but severe irAE. Risk factor assessment and patient education should be conducted before initiating ICIs therapy. If symptoms such as fever and hypotension occur during treatment, accompanied by abnormalities in characteristic indicators like C-reactive protein and related cytokines, individualized treatment should be implemented as early as possible to ensure the safe administration of ICIs.

## 1 Introduction

Immune checkpoint inhibitors (ICIs), a class of agents designed to specifically block negative immune regulatory targets such as programmed cell death protein 1 (PD-1) and its ligands(PD-L1), or cytotoxic T-lymphocyte-associated protein 4 (CTLA-4), are widely employed in first-line, second-line, and perioperative treatments for a broad spectrum of malignancies including lung cancer, melanoma, renal carcinoma, and gastric carcinoma. As a landmark advancement in tumor immunotherapy, ICIs can exert their antitumor effects by reversing immune suppression and enhancing T-cell-mediated immunity (1), which have progressively emerged as a novel therapeutic option in oncology in clinical practice in recent years. At present, multiple types of ICIs have been approved for marketing worldwide, consisting of PD-1/PD-L1 inhibitors, and CTLA-4 inhibitors. Among these, commonly applied agents represented by pembrolizumab, nivolumab, sintilimab, toripalimab and camrelizumab have demonstrated prominent survival benefits in the clinical management of various solid tumors (2).

With the widespread clinical application of ICIs, reports of corresponding immune-related adverse events (irAEs) have been increasingly frequent, among which cytokine release syndrome (CRS) stands as a representative irAE. Also known as “cytokine storm”, CRS refers to a systemic inflammatory response triggered by multiple stimuli including infections, pharmaceutical agents and chimeric antigen receptor T-cell (CAR-T) therapy, characterized by the abrupt massive aberrant release of cytokines such as tumor necrosis factor-alpha (TNF-α), interleukin-6 (IL-6), IL-8, IL-10, and interferon-gamma (IFN-γ) within a short period (3). Relevant research (4) has indicated that approximately 4.6% of patients receiving ICIs treatment may develop CRS. Moreover, even when CRS occurs in patients treated with ICIs, its clinical manifestations may be easily misdiagnosed as sepsis or other infectious conditions, thereby delaying appropriate treatment. To date, numerous cases of ICI-related CRS including fatal outcomes have been documented (5), therefore, it is imperative to enhance the awareness and recognition of CRS among healthcare professionals. On this basis, the present study adopts a literature review approach to summarize the incidence characteristics, clinical manifestations, monitoring biomarkers and management strategies of this irAE by collating published relevant case reports (including case series, similarly hereinafter), which is expected to provide references for the differential diagnosis of CRS and safe clinical administration of ICIs.

## 2 Data and methods

### 2.1 Literature search strategy

In this study, domestic and foreign databases such as China National Knowledge Infrastructure (CNKI), Wanfang Database(Wanfang), VIP Database(VIP), Chinese Medical Journal Network(CMJK), Web of Science(WoS), PubMed, and Embase were applied to comprehensively search and collect the case reports on ICIs-related CRS. The search strategy was a thematic pattern of “drug” AND “disease”, and the search keywords in this study should include their synonyms or synonymous expressions. That was to say, the term “drug” should cover the classifications and specific drugs of ICIs, while the term “disease” include cytokine release syndrome (CRS) and cytokine storm. Therefore, taking PubMed as an example, the specific retrieval strategy of this study was as follows: (“immune checkpoint inhibitor” OR “ICI” OR “programmed cell death protein 1” OR “PD-1” OR “anti-programmed death-L1” OR “PD-L1” OR “CTL activation antigen-4” OR “CTL-4”) AND (“cytokine release syndrome” OR “cytokine storm”).

To maximize the capture of relevant case reports, this study not only conducted a systematic search for cases associated with individual ICIs, but also manually screened the reference lists of the relevant adverse reaction reports or review articles. The search strategy for drug-related cases was as follows: (“toripalimab” OR “envafolimab” OR “sintilimab” OR “pembrolizumab” OR “nivolumab” OR “camrelizumab” OR “tislelizumab” OR “durvalumab” OR “atezolizumab” OR “ipilimumab” OR “penpulimab” OR “sugemalimab” OR “zimberelimab” OR “adberelimab” OR “pucotenlimab” OR “serplulimab” OR “retifanlimab” OR “cemiplimab” OR “cadonilimab” OR “avelumab” OR “tremelimumab”) AND (“cytokine release syndrome” OR “cytokine storm”). This study was last update on December 31, 2025.

### 2.2 Screening strategy

A systematic literature search was conducted to identify the eligible case reports by screening titles, abstracts, and full texts. Eligible cases were included if they met the following prespecified criteria: (1) written in both Chinese and English; (2) a confirmed diagnosis of CRS according to the American Society for Transplantation and Cellular Therapy (ASTCT) 2019 consensus grading criteria (6); (3) complete availability of patient demographic and clinical information; (4) documented exposure to ICIs; (5) clinical manifestations and laboratory findings consistent with the established CRS diagnostic criteria; (6) explicit exclusion of potential confounding conditions, including sepsis, hemophagocytic lymphohistiocytosis (HLH), and infectious etiologies, as reported in the case description.

At the same time, the following types of reports were excluded based on those criteria: (1) articles written in languages other than English or Chinese; (2) clinical trials, pharmacovigilance studies on adverse reactions, systematic or narrative reviews, and any literature that did not present individual case reports; (3) CRS attributable to etiologies other than ICIs, including but not limited to SARS-CoV-2 infection; (4) duplicate records or cases reported across different databases.

### 2.3 Retrospective analysis

The methodological quality of the included case reports was assessed by using the Critical Appraisal Checklists developed by The Joanna Briggs Institute (JBI), which were shown in **Tables 1**, **2**. Each criterion was scored as 1 point if the item was fully met; For case reports, studies with a total score of ≥7 were classified as high quality, those scored between 4 and 6 were considered moderate quality, while studies with a score of ≤3 were rated as low quality (7). As for the case series, a score of ≥ 8 was considered a high-quality study (7). In accordance with established methodological approaches for literature reviews, demographic data of included cases, ICIs administration, and information regarding the onset and management of CRS were extracted and compiled into Microsoft Office Excel 2021. Descriptive statistical analyses were subsequently performed to delineate the clinical characteristics and therapeutic implications of ICI-related CRS. The continuous variables were expressed as mean ± standard deviation, while the enumeration data were presented as percentages (%). For articles in which the CRS grade was not explicitly reported, the severity was determined according to the ASTCT 2019 consensus grading criteria (6).

**Table 1:** JBI critical appraisal checklist for case reports.

Criteria	Content	Criteria	Content
1	Were patient’s demographic characteristics clearly described?	5	Was the intervention(s) or treatment procedure(s) clearly described?
2	Was the patient’s history clearly described and presented as a timeline?	6	Was the post-intervention clinical condition clearly described?
3	Was the current clinical condition of the patient on presentation clearly described?	7	Were adverse events (harms) or unanticipated events identified and described?
4	Were diagnostic tests or assessment methods and the results clearly described?	8	Does the case report provide takeaway lessons?

**Table 2:** JBI critical appraisal checklist for case series.

Criteria	Content	Criteria	Content
1	Were there clear criteria for inclusion in the case series?	6	Was there clear reporting of the demographics of the participants in the study?
2	Was the condition measured in a standard, reliable way for all participants included in the case series?	7	Was there clear reporting of clinical information of the participants?
3	Were valid methods used for identification of the condition for all participants included in the case series?	8	Were the outcomes or follow up results of cases clearly reported?
4	Did the case series have consecutive inclusion of participants?	9	Was there clear reporting of the presenting site(s)/clinic(s) demographic information?
5	Did the case series have complete inclusion of participants?	10	Was statistical analysis appropriate?

The processes of literature retrieval, screening, data extraction, grading determination and quality assessment were independently conducted by two reviewers, with all discrepancies resolved through cross-verification to establish the final dataset.

## 3 Results

### 3.1 Literature results

**Figure 1:**
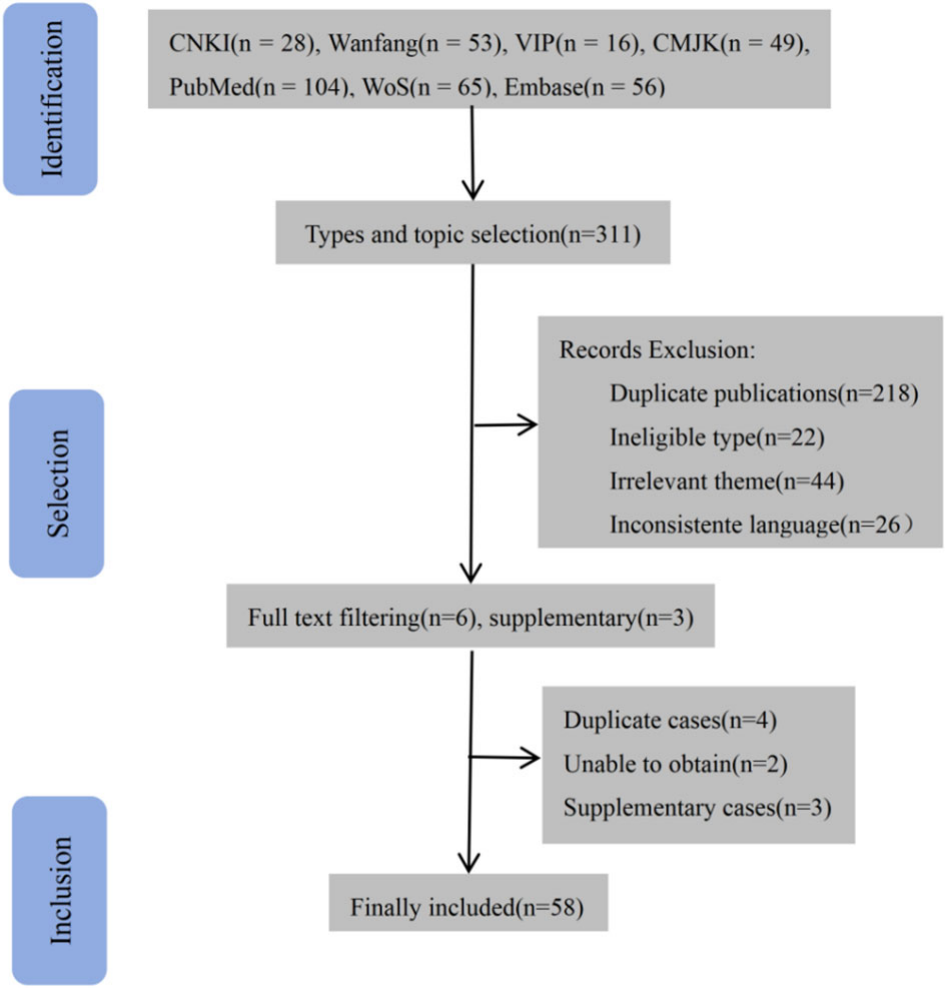
Screening process of this study.

**Figure 2:**
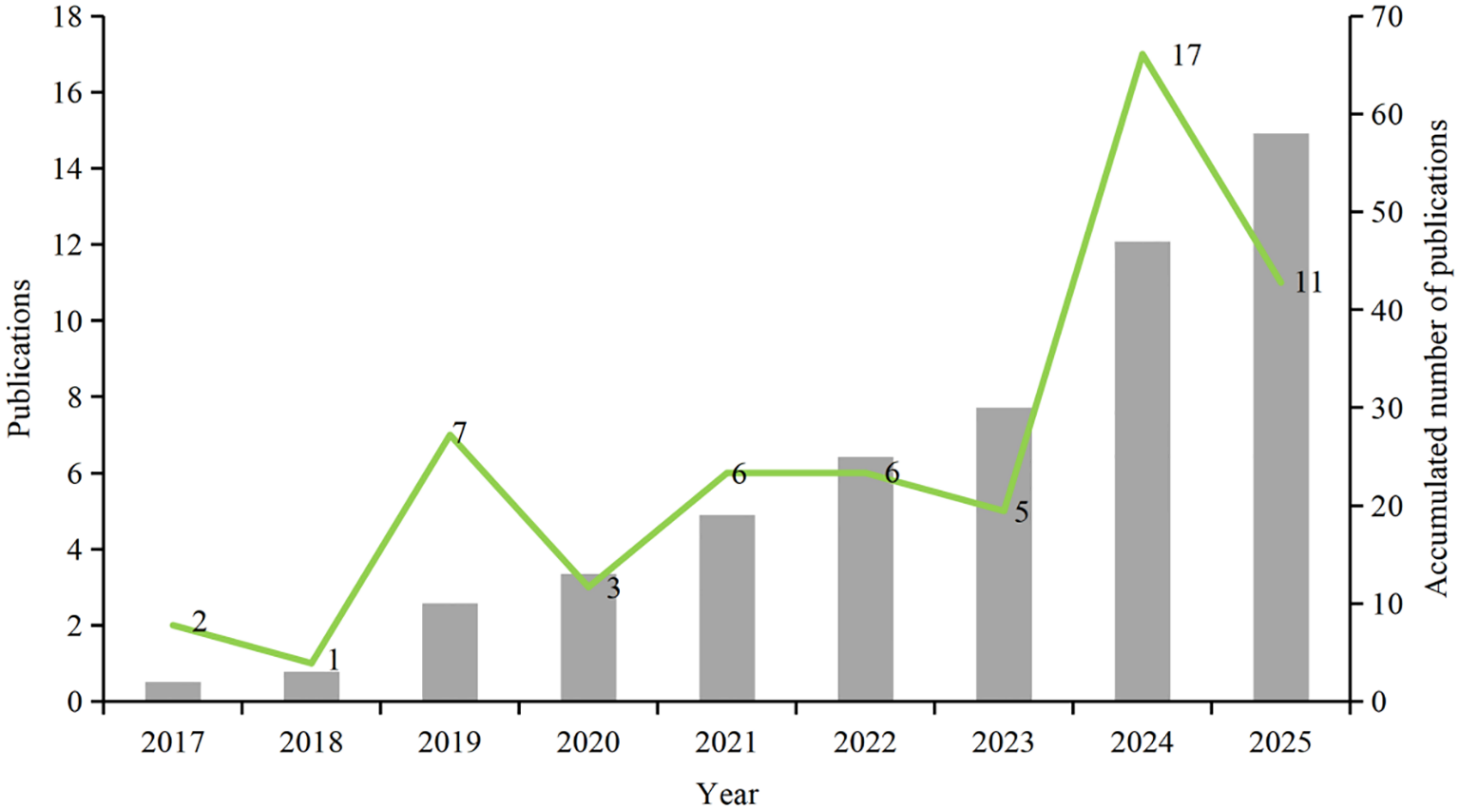
Annual publication volume.

The literature screening and selection process was shown in **Figure 1**. After initial screening and removal of duplicate publications and overlapping cases, 55 eligible studies were identified. An additional three relevant articles were obtained after manual screening of the reference lists of the retrieved literature. Ultimately, a total of 58 studies were finally included in this review, comprising 2 Chinese (8, 9) and 56 English (10–65) articles (including one case series). Notably, the earliest case report of CRS identified in this review was published in 2017, with the highest number of publications (17 articles) occurring in 2024, which was shown in **Figure 2**.

### 3.2 Results of literature quality appraisal

The results of methodological quality appraisal were presented as follows: 44 case reports scored ≥7 were thus categorized as high quality; 12 case reports were rated as medium quality, with only one case report being of low quality; while another case series was of high quality (scored = 8). In general, all included cases adequately described the demographic characteristics of the patients, medical histories, concomitant medications, and other adverse events. Furthermore, they provided comprehensive and detailed accounts of diagnostic assessments, therapeutic interventions, and clinical outcomes, rendering the included case reports suitable for evidence synthesis in case-summary analyses. What’s more, several moderate- and high-quality reports also offered informative clinical lessons and practical insights that might serve as a valuable reference for clinicians in real-world practice.

### 3.3 Basic information

As summarized in **Table 3**, a total of 59 patients were identified from the included articles, comprising 36 males (61.02%) and 23 females (38.98%). The patients ranged in age from 26 to 85 years (mean, 58.54 ± 15.29 years), and those aged ≥ 60 represented 54.24% of the cohort. Only 17 patients (28.81%) had predominantly age-related underlying comorbidities, such as hypertension (10 cases), hyperlipidemia (7 cases), and diabetes mellitus (6 cases). The clinical diagnoses encompassed 16 distinct tumor types, represented by lung cancer (including lung adenocarcinoma and lung squamous cell carcinoma; 26 cases), gastric carcinoma (5 cases), renal carcinoma (5 cases), and melanoma (5 cases). Geographically, these included cases originated from multiple countries in the world, with the highest contributions from Japan (23 cases), China (13 cases), and the USA (10 cases).

**Table 3:** Basic information of included cases.

Parameter	Classification	Frequency	Parameter	Classification	Frequency
Malignancy types	lung cancer	26	Sex	male	36
	gastric carcinoma	5		female	23
	renal carcinoma	5	Underlying comorbidities	not mentioned	34
	melanoma	5		hypertension	10
	hodgkin’s lymphoma	3		none	8
	cervical cancer	3		hyperlipidemia	7
	esophageal carcinoma	2		diabetes mellitus	6
	liver cancer	2		pancreatitis	2
	alveolar soft part sarcoma	1		heart disease	2
	hypopharyngeal cell carcinoma	1		asthma	2
	head & neck cancer	1		atopic dermatitis	1
	breast cancer	1		cholangitis	1
	skin cancer	1		arthritis	1
	colon cancer	1			
	cardia cancer	1			
	colorectal cancer	1			

### 3.4 Concomitant therapy and ICIs usage

**Figure 3:**
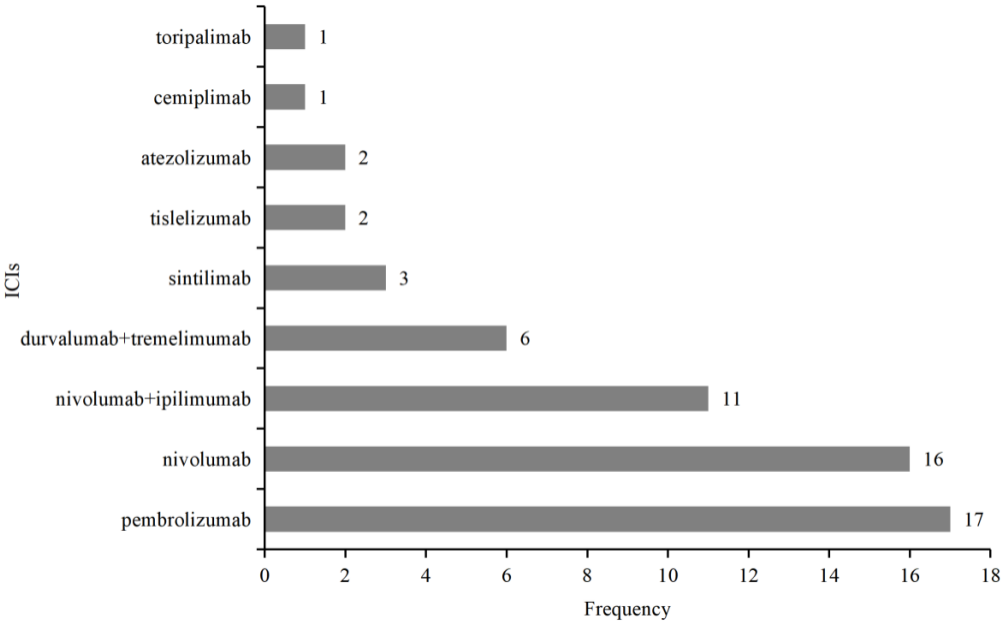
Information on ICIs usage.

A total of 34 patients (57.63%) received concomitant therapies in clinical practice, primarily pemetrexed + platinum-based drugs (11 cases) and paclitaxel + platinum-based drugs(5 cases). Across the treatment course, a total of 10 different ICIs were administered (shown in **Figure 3**). That is to say, 40 patients were treated only with PD-1 inhibitors, including pembrolizumab (17 cases), nivolumab (16 cases), sintilimab (3 cases), tislelizumab (2 cases), cemiplimab (1 case), and toripalimab (1 case). 17 patients received dual ICIs regimens, including nivolumab + ipilimumab (11 cases) and durvalumab + tremelimumab (6 cases); and anti-PD-L1 monotherapy with atezolizumab was administered to 2 patients.

In terms of the cycle of ICIs, the exact treatment cycle at the time of CRS onset was not specified in 5 cases. Among the remaining patients, CRS occurred during the first four treatment cycle in 43 cases (72.88%); while only 11 patients (18.64%) experienced CRS at or beyond the fifth cycle.

### 3.5 Information of adverse reactions

The adverse reaction profile was summarized in **Table 4**. According to the articles and the ASTCT 2019 consensus grading criteria, the severity grading of CRS comprised 22 cases with grade 1–2 and 37 cases with grade 3-4 (severe to critical). Prior to the confirmed diagnosis of CRS, all patients exhibited a variety of clinical symptoms, predominantly fever (49 cases), hypotension (27 cases), and fatigue (14 cases). Concurrently, 32 patients (54.24%) experienced at least one additional irAE, primarily including kidney injury (11 cases), pneumonia (8 cases), HLH (5 cases), and hepatitis (4 cases).

**Table 4:** Information of adverse reactions.

Parameter	Classification	Frequency	Parameter	Classification	Frequency
Inflammatory indicators	IL-6	35	Severity	grade 1	5
	IL-10	10		grade 2	17
	TNF-α	9		grade 3	18
	IFN-γ	9		grade 4	19
	IL-8	8	Biochemical Indicators	C-reactive protein	46
	IL-2	5		ferritin	21
	IL-1β	4		white blood cells	19
	IL-17	2		platelets	17
	IL-9	1		neutrophils	8
	IL-7	1		lymphocytes	5

As noted in the ASTCT 2019 consensus criteria, ICI-related CRS was frequently associated with organ damage involving the lungs and kidneys. In the present cohort, the results of pulmonary imaging were mainly normal (18 cases), ground-glass opacity (5 cases) and pulmonary edema (5 cases). Common biochemical indicators in this study involved C-reactive protein (CRP, 46 cases), ferritin (21 cases), white blood cells (19 cases), and platelets (17 cases); while the predominant abnormal inflammatory indicators were IL-6 (35 cases), IL-10 (10 cases), TNF-α (9 cases), and IFN-γ (9 cases).

### 3.6 Treatment and outcomes

The management strategies for CRS consisted of temporary or permanent discontinuation of ICIs, glucocorticoid administration, additional immunosuppressive agents, and other symptomatic supportive treatment, such as anti-infective treatment, nutritional support, antipyretic therapy, and fluid replacement (66). The major measures of enrolled cases were categorized as follows: glucocorticoids only (10 cases), glucocorticoids + immunomodulators (9 cases), glucocorticoids + immunomodulators + organ support + injury treatment (7 cases), glucocorticoids + immunomodulators + organ support (6 cases).

Following these comprehensive intervention, the condition of 50 cases ultimately improved. Notably, 3 patients experienced recurrence of CRS after initial improvement, but 2 of these recurrent cases died due to ineffective treatment. These observations underscored the generally poor prognosis and high mortality associated with ICI-related CRS, warranting heightened clinical awareness and prompt, aggressive management.

## 4 Discussion

### 4.1 Clinical characteristics

CRS is a phenomenon of immune hyperactivation during ICIs therapy, characterized by a systemic inflammatory syndrome resulting from the massive and abrupt release of cytokines. In this study, the male-to-female ratio among included cases was approximately 1.57:1, with a slight predominance of males; to some extent suggesting that males may constitute a high-risk population for ICI-related CRS. On the one hand, this distribution is partly attributable to gender disparities in tumor incidence; as male gender is a well-established contributing factor for non-small cell lung cancer (67). On the other hand, male patients tend to have a lower baseline state of immune suppression compared to females. Given the widespread application of ICIs in elderly populations with a high baseline incidence of malignancies, immune homeostasis in elderly male patients is more susceptible to disruption, thereby leading to the occurrence of various irAEs, including CRS. Although existing study has indicated that ICI-related irAEs predominantly occur in elderly patients (68), while the age range of cases in this study spanned from young and middle-aged adults to the elderly, there was no significant difference in the proportion between these age groups. Therefore, the relationship between the occurrence of ICI-related CRS and age factors requires to be further validated with large-sample data. Meanwhile, the clinical diagnoses of enrolled patients involved a wide range of solid malignancies, yet most patients had no specific circumstances regarding combination medication or underlying medical history. It can be inferred that ICI-related CRS may be an intrinsic drug-related irAE independent of tumor type or patient demographics, which aligns with the findings of Pichler WJ (69). The majority of cases in our study predominantly originated from Japan, China, and USA, with lung cancer being the most common primary malignancy. These clinical characteristics are generally in line with the results reported by Xi X et al. (70), which also indicates that ICI-related CRS is more prevalent in male patients suffering from lung cancer, melanoma; and higher tumor burden status correlates with an increased probability of developing CRS. Consequently, comprehensive assessment of patients’ tumor burden status is essential prior to ICIs initiation to stratify their risk of developing CRS.

CRS is only labeled as a rare irAE in a limited subset of ICIs package inserts. For instance, CRS has been reported only during the clinical trial phase of tislelizumab; although grade ≥3 cases occurred, the overall incidence of this irAE remained less than 0.1%. It indicates that PD-1 inhibitors are more likely to induce CRS compared to PD-L1 inhibitors in the monotherapy setting, and pembrolizumab and nivolumab account for the majority of ICI-related CRS. On the one hand, PD-1 inhibitors exhibit a broader scope of pharmacological effects by simultaneously blocking both the PD-L1 and PD-L2 signaling pathways, in contrast to PD-L1/CTLA-4 inhibitors (71). On the other hand, agents such as pembrolizumab and nivolumab are approved earlier than other PD-1 inhibitors (e.g., sintilimab, tislelizumab) and thus have been more widely administered both domestically and internationally. From a mechanistic perspective, these later-approved PD-1 inhibitors do not differ fundamentally from pembrolizumab or nivolumab, suggesting that clinicians should remain vigilant for CRS when prescribing these newer agents. Previous meta-analyses (72) has demonstrated that compared with PD-1/PD-L1 inhibitors alone, the combination of PD-1/PD-L1 inhibitors with CTLA-4 inhibitors can synergistically enhance immunotherapeutic effects through distinct but complementary pathways, which also inevitably elevates the risk of irAEs. Among the included subjects, 17 patients (28.81%) developed CRS following combined immunotherapy. Excluding five cases with unspecified treatment cycles, approximately 80% of CRS events occurred within the first four cycles of ICIs administration, and nearly one-third of patients experienced CRS during the first cycle of treatment. This temporal distribution indicates that ICI-related CRS is likely driven by rapid immune activation, which provides critical clues for the clinical diagnosis of this irAE.

### 4.2 Indicators and manifestations

Au L et al. (73) have highlighted that elevated inflammatory markers, thrombocytopenia, abnormal cytokine levels, and responsiveness to glucocorticoids hold significant clinical value for confirming the diagnosis of CRS. In these enrolled cases, abnormal inflammatory indicators were predominantly characterized by elevations in IL-6, IL-10, TNF-α and IFN-γ. Among these, IL-6 is a pivotal cytokine mediating fever, driving the acute-phase elevation of CRP, and contributing to potential vascular leakage in CRS patients. Previous research (74) has demonstrated that the severity of CRS is inextricably linked to *in vivo* IL-6 levels; therefore, real-time monitoring of IL-6 fluctuations aids in determining the optimal timing for administration of its inhibitors such as tocilizumab. The vast majority of patients in this study presented elevated IL-6, and this high detection rate in real-world clinical cases further highlights the close association between IL-6 and ICI-related CRS. Concurrently, most cases also exhibited elevations in IL-10, TNF-α, and IFN-γ, which not only confirms that the pathogenic mechanism of ICI-related CRS is consistent with the general pathophysiological process of CRS, but also provides direct theoretical rationale for the targeted blockade of CRS using cytokine inhibitors like tocilizumab and infliximab. As an acute-phase reactant, CRP is predominantly synthesized by hepatocytes in response to IL-6 stimulation. Thus, CRP can serve as a reliable surrogate indicator of IL-6 biological activity within a certain range (75). Consequently, relevant scholars have proposed that CRP can be adopted as a measurable indicator to evaluate CRS severity, noting that a CRP level ≥200 mg/L confers 100% specificity (76). In addition, ferritin functions not only merely as an inflammatory marker, but also as critical evidence of macrophage activation when presenting at abnormal levels. Collectively, these abnormal inflammatory and biochemical abnormalities observed in the included cases constitute a comprehensive laboratory assessment panel that may facilitate the clinical diagnosis and monitoring of ICI-related CRS, which offers valuable references for future CRS surveillance, diagnosis, and severity stratification.

Although the included cases presented with various initial symptoms, fever, hypotension and fatigue were the predominant manifestations, which was consistent with previous research findings. As reported by Remon J et al. (77), CRS is mostly initiated by fever and may further progress to severe organ failure, shock or disseminated intravascular coagulation, with hypoxia and pulmonary edema serving as prominent markers of advanced CRS. Fever in these patients is primarily caused by the rapid and massive release of pyrogenic cytokines including IL-6, IL-1 and TNF-α into the circulation. Concurrently, altered vascular permeability and vasodilation can lead to hypotension, indicating a high risk of deterioration to higher-grade CRS. This excessive immune-inflammatory response mediated by ICIs triggers the secretion of multiple effector cytokines, including IL-6, IL-10, IFN-γ, and TNF-α, resulting in endothelial complement activation, coagulation cascade dysregulation, and changes in capillary permeability, which can ultimately culminate in multi-organ dysfunction (78). Moreover, over half of the patients with ICI-related CRS concurrently suffered at least one other organ-specific irAE, including renal injury, pneumonia, HLH, and hepatitis, all of which were well-recognized as severe irAEs induced by excessive immune activation. Notably, the clinical manifestations and abnormal biochemical parameters of primary HLH closely resemble those of severe CRS (79), and considering that some patients with CRS may also develop concurrent HLH as well, it is crucial to differentiate them during ICI therapy. The core clinical manifestations of CRS are fever and hypotension, with IL-6 as the key driving cytokine, whereas HLH is characterized by pancytopenia accompanied by markedly elevated ferritin levels (80, 81).

### 4.3 Treatment and management

At present, there are no dedicated diagnostic criteria or therapeutic guidelines specifically issued for ICI-related CRS, but the management of this irAE can draw reference from the established guidelines for CRS induced by CAR-T immunotherapy (82, 83). The ASTCT has stratified CRS into four severity grades based on patients’ clinical manifestations to guide clinical treatment (6), therefore, the treatment of ICI-related CRS requires multidisciplinary comprehensive intervention tailored to clinical demands, as well as individualized regimens formulated according to the distinct patient manifestations and CRS grades. That is to say, For patients with grade 1–2 CRS, supportive measures including antipyretics, oxygen therapy, and fluid replacement should be initiated following an initial assessment, with careful attention to differential diagnoses such as infection and capillary leak syndrome; when necessary, glucocorticoids and immunomodulators may be supplemented. Conversely, the management of grade 3–4 patients should occur in an ICU admission, combined with targeted interventions such as glucocorticoids, immunomodulators as well as support and injury treatment in accordance with individual conditions (84). The combination of therapeutic approaches adopted in the included cases reflects this graded approach: monotherapy with glucocorticoids or supportive treatment alone appears more suitable for grade 1–2 CRS or those without severe organ impairment, whereas combination therapies are primarily employed for critically severe cases. Cytokine inhibitors such as tocilizumab and infliximab serve as core therapeutic agents by targeted blockade of the central pathways underlying CRS. Concurrent administration of organ-supportive measures (vasopressors, mechanical ventilation) realizes an integrated strategy of etiological therapy and organ function replacement.

Although most enrolled patients achieved favorable outcomes following aggressive intervention, a subset of cases also experienced clinical deterioration, including fatal cases unresponsive to therapy after recurrence. Further statistical analysis revealed that the primary malignancies among deceased patients were predominantly lung cancer and melanoma, the majority presented with grade 3-4, and nivolumab was the most commonly administered. Collectively, primary tumor type and CRS severity may confer an elevated risk of mortality. In particular, CRS with a severity of 3–4 is inherently associated with higher mortality rates. As pointed out by Nie J et al (3), greater CRS severity corresponds to an increased risks of organ dysfunction, hemodynamic instability and multiorgan failure; hence, the breadth and severity of organ involvement directly contribute to the increased fatality rate. Meanwhile, lung cancer has been validated as a predictive factor for severe or fatal irAEs (85). Nevertheless, there is no evidence to indicate that nivolumab can carries a higher lethal potential compared with other ICIs, such as PD-1/PD-L1 inhibitors. And the predominance of nivolumab use among the deceased cases in this study more likely reflects its widespread utilization in clinical practice.

Given the increasing administration of ICIs, it is imperative to implement predictive monitoring and proactive management of CRS in clinical practice. As noted by Si S et al. (86), CRS is an antigen-nonspecific toxicity; hence, targeted management of this irAE should be implemented according to the core factors including disease severity and underlying etiologies. First, the management and monitoring of CRS must be a dynamic process spanning the entire course of ICI administration, the relevant clinical symptoms and early warning biomarkers, with preventive assessments conducted in advance. For instance, high-risk patients should undergo targeted evaluations of tumor burden, baseline inflammatory profiles, and biochemical parameters. Upon suspected or confirmed CRS diagnosis, interventions and therapeutic measures should adhere to the principles of “graded treatment, precision targeting, and concurrent anticancer efficacy.” Regarding pharmacological selection, although tocilizumab has been approved by FDA for treating severe or life-threatening CRS, a review of medication usage in the included cases revealed that some patients showed no significant response to it (87). Accordingly, future strategies should select appropriate immunomodulators based on accurate cytokine subtyping to realize precision treatment for CRS. In addition, it is crucial to differentiate and manage other concomitant irAEs during CRS treatment.

Although ICI-related CRS, CAR-T-related CRS, sepsis and HLH share overlapping typical manifestations including fever, hypotension and pulmonary edema in clinical practice, there exist fundamental discrepancies between them in multiple dimensions including core pathogenesis and triggering mechanisms. As exemplified by sepsis, the cornerstone for confirming a diagnosis of it lies in identification of an infectious source (88); while the pathogenetic mechanism underlying CAR-T-related CRS is entirely distinct from that of ICI-related CRS. It arises from the rapid proliferation of infused CAR-T cells upon recognition of target antigens, which leads to robust secretion of abundant proinflammatory cytokines, with monocytes and macrophages further amplifying this inflammatory cascade (89). CAR-T-related CRS typically manifests in the early phase after CAR-T cell infusion, and its inflammatory cascade is predominantly driven by IL-6 (90). In addition to identifying the similar adverse reactions, rechallenge therapy for patients who have achieved clinical remission remains a central clinical concern. it is well known that dual immunotherapy combination, CRS severity, high tumor burden, and a prior history of irAEs may be highly correlated with CRS recurrence following ICIs rechallenge. Therefore, a comprehensive risk-benefit assessment balancing anti-tumor efficacy and toxicological risks is mandatory prior to ICI rechallenge. In patients who experienced mild CRS, ICI resumption may be considered under close surveillance; whereas severe or life-threatening irAEs generally warrant permanent discontinuation of ICIs (91, 92).

This study summarized the core issues including the incidence characteristics, clinical manifestations and therapeutic interventions of ICI-related CRS based on published case reports, which can offer valuable insights for the safe clinical administration of ICIs and the standardized management of CRS. Due to the fact that this study is a retrospective literature-based analysis, some of the conclusions drawn above may have certain limitations. For example, incomplete literature retrieval and delayed case data may reduce the universality of the analytical results. Meanwhile, discrepancies exist in clinicians’ awareness of CRS, and individualized variations are also observed in their therapeutic regimens. Future efforts should continuously focus on ICI-related CRS to fully and accurately characterize the clinical profiles of this irAE, so as to furnish more evidence for safe ICI application in clinical practice.
